# A rare complication of biliary stent insertion: biliary stent migration causing jejunal perforation

**DOI:** 10.1093/jscr/rjag286

**Published:** 2026-04-25

**Authors:** Su Su (Shannon) Naing

**Affiliations:** Department of General Surgery, The Hervey Bay Hospital, Cnr Nissen Street, &, 141/169 Urraween Rd, Pialba QLD 4655, Australia

**Keywords:** stent migration, ERCP, complications of ERCP

## Abstract

Biliary stent placement is a common therapeutic intervention for biliary obstruction during endoscopic retrograde cholangiopancreatography. While generally safe, migration of these stents can occur. Distal migration leading to small bowel perforation is an exceptionally rare and life-threatening complication. We present the case of a 67-year-old female with a history of complex biliary disease and recent stent placement who presented with acute lower abdominal pain and septic shock. Computed tomography revealed a jejunal perforation caused by a migrated biliary stent. The patient underwent emergency exploratory laparotomy and small bowel resection. This case highlights the importance of maintaining a high index of suspicion for stent-related complications in patients presenting with an acute abdomen following biliary instrumentation, even months after the initial procedure.

## Introduction

Biliary stent placement during endoscopic retrograde cholangiopancreatography (ERCP) is a well-established therapeutic intervention for the relief of biliary obstruction. While the procedure is generally safe, it is associated with several adverse events, including pancreatitis, hemorrhage, perforation, and cholangitis. Stent migration is a recognized complication, reported in ~5%–10% of cases [1]. While most migrated stents pass spontaneously through the gastrointestinal tract, distal migration can rarely lead to critical complications such as intestinal obstruction, hemorrhage, and perforation. Jejunal perforation is particularly uncommon, with ˂1% of migrated stents causing bowel perforation [2]. This report discusses the management of a patient presenting with jejunal perforation secondary to a migrated biliary stent.

## Case presentation

A 67-year-old female presented to the Emergency Department with an acute onset of severe lower abdominal pain and septic shock. Her past medical history included cardiomyopathy, atrial fibrillation, asthma, type 2 diabetes mellitus, dyslipidaemia, and hypothyroidism.

Earlier in the year, the patient had been treated for gallstone pancreatitis with choledocholithiasis, requiring ERCP. During the index procedure, a 5 Fr × 5 cm plastic stent was placed into the ventral pancreatic duct, and a 10 Fr × 7 cm plastic stent was placed into the common bile duct. Two months later, she underwent a repeat ERCP for the removal of the pancreatic duct stent, followed by an interval laparoscopic cholecystectomy.

**Figure 1:**
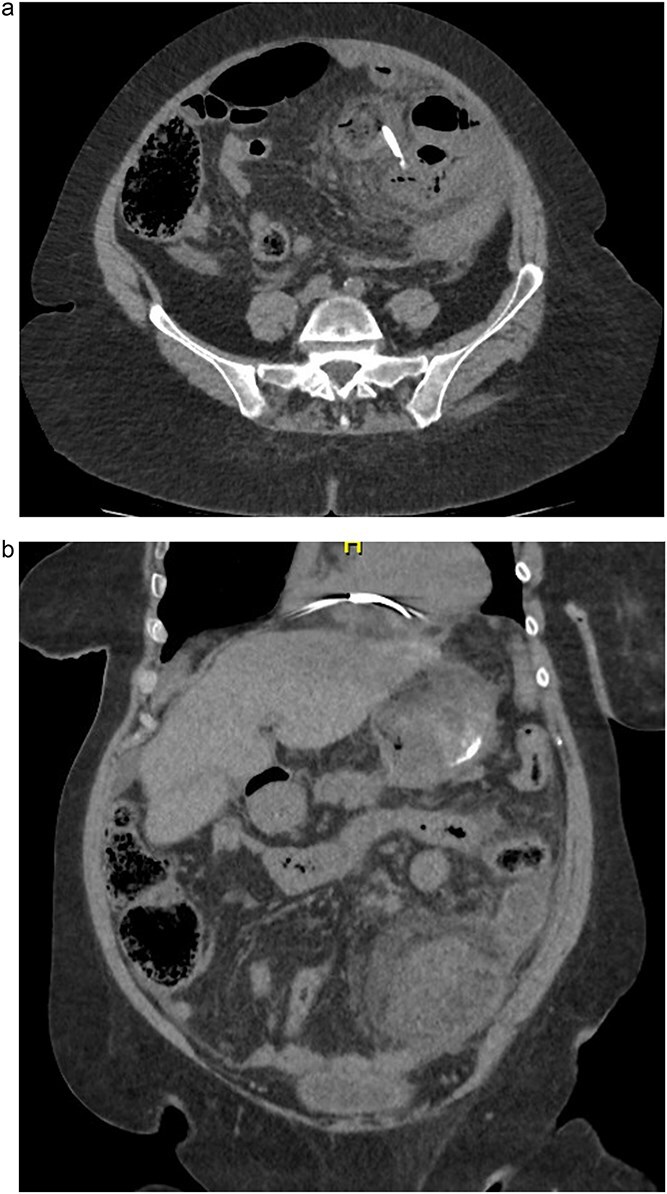
(a) CT scan of the abdomen and pelvis. Axial view showing a linear hyperdense foreign body within the small bowel loop, consistent with the migrated biliary stent. (b) CT scan of the abdomen and pelvis. Coronal view revealing an 8 cm fluid collection with rim enhancement adjacent to the jejunum, indicative of an intra-abdominal abscess secondary to perforation.

**Figure 2:**
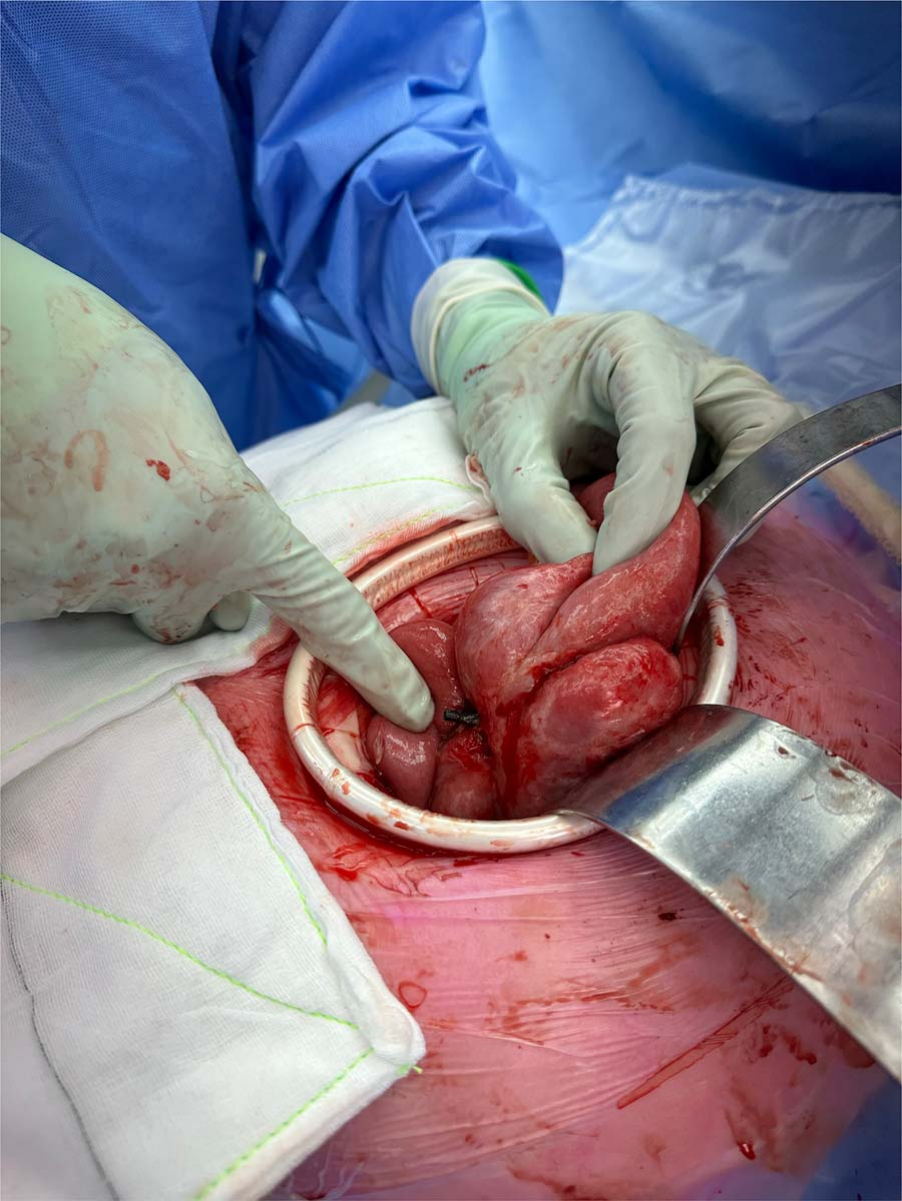
Intraoperative photograph demonstrating the biliary stent eroding through the wall of the jejunum. Note the surrounding inflammation and purulent exudate consistent with perforation and local peritonitis.

On presentation to the Emergency Department, the patient was hemodynamically unstable. Computed tomography (CT) imaging of the abdomen and pelvis demonstrated small bowel obstruction and a linear foreign body consistent with a biliary stent perforating the jejunum (Fig. 1a) The CT also revealed the 8 cm abscess cavity adjacent to the perforated jejunum (Fig. 1b). The patient was taken for emergency exploratory laparotomy. Intraoperative findings revealed a large volume of purulent and enteric free fluid within the peritoneal cavity. The biliary stent was found to have perforated the small bowel, with an adjacent chronic abscess cavity measuring 8 cm (Fig. 2).

A small bowel resection with washout was performed. The patient recovered uneventfully post-operatively.

## Discussion

ERCP is the gold standard for managing biliary obstruction, yet it carries a risk of complications ranging from 5% to 10% [3]. While post-ERCP pancreatitis and hemorrhage are well-recognized, biliary stent migration is a distinct complication that presents unique diagnostic and therapeutic challenges. Migration can occur proximally (into the biliary tree) or distally (into the duodenum). Distal migration is reported in ~3%–6% of cases [4]. Although most distally migrated stents pass spontaneously through the gastrointestinal tract without sequelae, ˂1% result in bowel perforation [5].

The pathophysiology of perforation typically involves the impaction of the stent against the bowel wall, leading to pressure necrosis and subsequent erosion. While the duodenum is the most common site of perforation (specifically the C-loop) due to its fixed retroperitoneal position and proximity to the biliary tree, jejunal perforation, as seen in this case, is relatively rare [6]. Risk factors for distal migration and subsequent perforation include the use of straight plastic stents (as opposed to double-pigtail stents), larger diameter stents (>10 Fr), and patient-specific factors such as abdominal hernias, diverticula, or intra-abdominal adhesions from prior surgeries [4, 7].

In this case, the patient had a 10 Fr straight plastic stent. Literature suggests that straight polyethylene stents are more prone to migration than pigtail stents, which have coiled ends that serve as an anchoring mechanism [7]. Furthermore, the presence of an 8 cm abscess cavity adjacent to the perforation site suggests a subacute process where the stent eroded through the jejunal wall over time, initially contained by the omentum or adhesions, before rupturing and causing generalized peritonitis and septic shock. This highlights the variable clinical presentation of stent perforation, which can range from asymptomatic migration to acute abdominal catastrophe.

Diagnostic delay is common because symptoms often mimic other post-ERCP complications or unrelated abdominal pathology. Plain abdominal radiography can identify the position of the stent but often fails to detect free air or fluid. CT remains the imaging modality of choice, offering high sensitivity for detecting the stent’s precise location, extraluminal air, and abscess formation [8].

Management strategies depend on the patient’s clinical status and the location of the stent. If the stent has migrated but remains within the bowel lumen without perforation, endoscopic retrieval or conservative management with serial imaging is appropriate. However, once perforation occurs, surgery is almost invariably required. While laparoscopic retrieval and repair have been described for stable patients, exploratory laparotomy is indicated for patients presenting with hemodynamic instability or generalized peritonitis, as was necessary for this patient [9].

This case emphasizes the importance of vigilance regarding ‘forgotten’ or retained biliary stents. Although the patient underwent a repeat ERCP for pancreatic stent removal, the biliary stent migrated and subsequently caused severe morbidity. It serves as a reminder that even after the primary biliary pathology is addressed, the hardware used for treatment poses a continued risk until it is retrieved or passes.

## Conclusion

While most migrated biliary stents pass spontaneously or can be retrieved endoscopically, a subset can lead to serious complications such as perforation and peritonitis, necessitating emergency laparotomy. Therefore, in patients presenting with an acute abdomen and fever post-ERCP, a high index of suspicion for stent-related complications should be maintained. Early use of CT imaging is crucial for diagnosis and guiding surgical intervention.
